# Protective effect and mechanism of Sufentanil on acute lung injury in septic mice

**DOI:** 10.3389/fphar.2024.1514602

**Published:** 2025-01-16

**Authors:** Hongqiao Hou, Bowen Jiang, Aiqing Zhu, Junjun Hou, Zhe Qu, Ruping Liu, Aiqun Li

**Affiliations:** ^1^ Emergency Surgery Department, Yantai Affiliate Hospital of Binzhou Medical University, Yantai, Shandong, China; ^2^ Department of Dermatology and Venereology, Yantai Affiliate Hospital of Binzhou Medical University, Yantai, Shandong, China; ^3^ Department of Respiratory and Critical Care Medicine, Yantai Affiliate Hospital of Binzhou Medical University, Yantai, Shandong, China

**Keywords:** Sufentanil, sepsis, acute lung injury, janus kinase 2, STAT3 transcription factor, inflammation, apoptosis

## Abstract

This study was designed to investigate the protective effect and mechanism of Sufentanil on acute lung injury in septic mice based on network pharmacology and animal experiments, and to provide new ideas for clinical treatment. To this end, a protein-protein interaction (PPI) network for common targets was first constructed with Swiss Target Prediction Database, GeneCards Database, Draw Venn Diagram Software, STRING 11.5 Database, Cytoscape 3.10.0 Software and Metascape Database, and then key targets were subject to enrichment analysis by Gene Ontology (GO) and Kyoto Encyclopedia of Genes and Genomes (KEGG) to obtain the key targets of Sufentanil for the treatment of pulmonary sepsis, and then verified by animal experiments. A sepsis model was constructed by cecal ligation and puncture (CLP) in this study, and lung tissues and bronchoalveolar lavage fluid (BALF) were taken from each group of mice. The morphological changes of lung tissues and apoptosis were observed by HE and TUNEL staining, the content of inflammatory factors in the lung tissues was detected by ELISA, and the expression of proteins, such as p-JAK2 and p-STAT3, was detected in the lung tissues by Western blotting. According to the results of network pharmacology, a total of 40 common targets of were screened out for Sufentanil and pulmonary sepsis, and GO enrichment analysis involved 1,483 biological processes (BPs), 84 cellular components (CCs) and 125 molecular functions (MFs); KEGG enrichment analysis identified 137 signaling pathways with p < 0.05 such as JAK-STAT. According to the results of animal experiments, compared with the control group, mice in the model group had severe lung tissue injury and elevated expression of relevant inflammatory factors in lung tissue. Compared with the model group, CLP + Sufentanil group showed reduced pathomorphologic lesions, lower expression of inflammatory factors and apoptosis level, as well as lower expression of p-JAK2 and p-STAT3 proteins in lung tissue. The results of animal experiments were consistent with network pharmacology. In summary, Sufentanil may improve lung injury in septic mice by inhibiting the JAK2-STAT3 signaling pathway, which provides a basis for research on the mechanism of Sufentanil on pulmonary sepsis and clinical treatment.

## 1 Introduction

Sepsis is a systemic inflammatory response syndrome (SIRS) caused by infection and is a life-threatening organ dysfunction resulting from a dysregulated inflammatory response of the host to various pathogenic microorganisms that invade the organism (Singer et al., 2016; Driessen et al., 2018). It is also a common complication of a wide range of clinical conditions (Balk, 2014). There are approximately more than 30 million cases of sepsis each year (Fleischmann et al., 2016), and its mortality rate is as high as 20%–45% in intensive care units (Huang et al., 2019). Acute lung injury (ALI) is induced by various pathogenic factors such as pneumonia, infection and trauma, which result in structural and functional alterations of pulmonary capillary endothelial cells and alveolar epithelial cells, leading to diffuse interstitial and alveolar edema of the lungs (Bellani et al., 2016). The susceptibility of the lungs makes ALI one of the most common organ dysfunctions in sepsis, although recent research on the mechanisms of ALI caused by sepsis has achieved significant progress (Wang et al., 2024; Wang et al., 2022; Liu et al., 2022; Wang et al., 2024; Wang et al., 2022; Liu et al., 2022). Therefore, Research into effective drugs to reduce patient mortality is essential.

Inflammation and apoptosis are key factors in acute lung injury caused by sepsis, and they interact and together drive the progression of ALI (Chen et al., 2024; Xu and Wang, 2020; Wang et al., 2023). The inflammatory response leads to the release of a large number of cytokines, which in turn triggers the apoptosis of alveolar epithelial cells, and the increase of apoptotic cells further aggravates the inflammatory response, forming a vicious cycle (Chopra et al., 2009; Chen et al., 2021). Therefore, therapeutic strategies targeting inflammation and apoptosis may provide new avenues for treatment. Sufentanil is an opioid receptor agonist commonly used in anesthesia and in critically ill patients. In addition, sufentanil used in patients with sepsis can reduce pain and discomfort in patients, as well as meet sedation needs during assisted mechanical ventilation (Dinse et al., 2022). However, recent studies have shown that sufentanil shows positive effects in terms of anti-inflammatory and anti-tumor effects. Sufentanil has been shown to reduce the expression of nitric oxide synthase (iNOS), interleukin-6 (IL-6) and other inflammatory factors in myocardial ischemia-reperfusion (I/R) injury (Qiu et al., 2022). In another study, Sufentanil alleviated H/R-induced apoptosis, mitochondrial membrane potential dysfunction, oxidative stress, and inflammation through multiple pathways (Lu et al., 2022; Liu et al., 2023). In addition, sufentanil inhibits proliferation, invasion, epithelial-mesenchymal transformation (EMT), and inflammatory responses of breast cancer cells, while inducing apoptosis of these cells (Li et al., 2023). These results suggest that sufentanil plays an important role in anti-inflammatory and anti-apoptosis. In addition, through the analysis of network pharmacological studies, we found that sufentanil is closely associated with the JAK2-STAT3 signaling pathway in sepsis associated lung injury. Jak2-stat3 signaling pathway is one of the inflammatory pathways involved in the pathogenesis of lung injury. Inhibition of JAK2/STAT3 signaling pathway can reduce lung inflammation and apoptosis and protect mice from acute lung injury (Lou et al., 2022; Hu et al., 2022; Gao et al., 2021). However, the role of sufentanil in lung tissue injury in sepsis mice by regulating the JAK2-STAT3 signaling pathway remains unclear.

Therefore, in order to explore the mechanism of action and protective effect of sufentanil on acute lung injury in sepsis, this study established a mouse CLP model to study the potential molecular mechanism of sufentanil’s inflammation and apoptosis related to the JAK2-STAT3 signaling pathway, hoping to provide potential targets and new research ideas for the treatment of acute lung injury in sepsis.

## 2 Materials and methods

### 2.1 Reagent

Paraformaldehyde, HE staining kit, and Masson trichrome staining solution were all obtained from Solabio Biotechnology Co., Ltd. in Tongzhou District, Beijing, China. Anhydrous ethanol, TUNEL kit, and DMSO were obtained from Sinopharm Chemical Reagent Co., Ltd. in Xicheng District, Beijing, China. Neutral resin was purchased from Shanghai Aladdin Biochemical Technology Co., Ltd.

### 2.2 Animal study

A total of 40 BALB/C mice (18–24 g) were purchased from Jinan Pengyue Experimental Animal Breeding Co., Ltd. (Jinan, China). and acclimatized in polycarbonate cages with temperature (23°C ± 2°C), humidity (25 ± 5), and a 12-h light-dark cycle for 1 week prior to the experiments. During this period, experimental mice had free access to feed and water, and animal experiments were performed according to guidelines (Couto and Cates, 2019; Jones-Bolin, 2012). Animal experiments were conducted in accordance with the guidance of the Animal Experiment Ethics Committee of Binzhou Medical College.

### 2.3 Establishment of septic ALI model

A cecal ligation and puncture (CLP) mouse model was established according to the methods of the predecessors and guidelines (Li et al., 2019; Drechsler and Osuchowski, 2021; Standage et al., 2017), and mice were anesthetized by intraperitoneal injection of pentobarbital sodium 50 mg/kg (Hu et al., 2020). After anesthesia, the hair was shaved off in the abdomen of the mice and a 2-cm incision was made in the middle abdomen to expose the cecum. The cecum was ligated below the ileocecal valve with 3–0 suture, and then punctured with sterile 20-gauge needle. A small amount of feces was extruded, and then the cecum was restored to its position. The abdominal incision was closed in layers. Mice in sham-operated (Sham group) underwent the same laparotomy, i.e., only the cecum was exposed, but no cecal ligation or puncture was performed, and the same suture method was used. All mice had free access to water and feed after CLP. There were no abnormal deaths in the experiments. 24 h after CLP, all mice were euthanized by intraperitoneal injection of pentobarbital sodium 200 mg/kg (Wu et al., 2019a; Hu et al., 2017). The criteria for confirmation of euthanasia were complete cardiac arrest and dilated pupils. Lung tissues were then collected for further experiments. No abnormal deaths occurred during the experiment.

### 2.4 Grouping and drug administration

A total of 40 mice were randomly divided into 4 groups. i) Sham group (n = 10): laparotomy was performed without ligature or puncture, and saline 1 mL was injected into the caudal vein 30 min prior to surgery. ii) Sham + Sufentanil group (n = 10): laparotomy was performed without ligature or puncture, and Sufentanil (3 μg/kg, 1 mL) was injected into the tail vein 30 min prior to surgery. iii) CLP group (n = 10): CLP was performed, and saline 1 mL was injected into the caudal vein 30 min prior to surgery. iv) CLP + Sufentanil group: CLP was performed, and Sufentanil was injected into the tail vein 30 min before surgery. The experimental drug dose and the time of administration were selected according to previous experiments and guidelines (Hu et al., 2020; Zang et al., 2022). Sufentanil was purchased from Yichang Humanwell Pharmaceutical co. (Yichang, China).

### 2.5 Network pharmacology procedures

**FIGURE 1:**
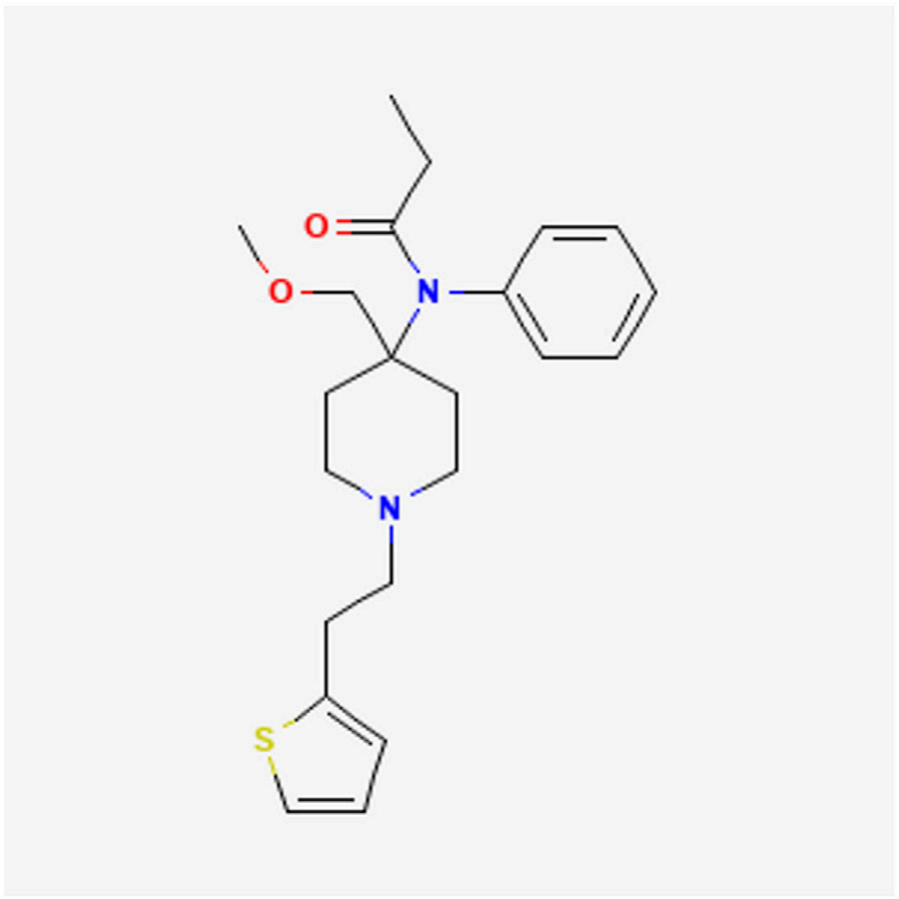
The 2D structure of sufentanil.

The relevant predicted targets for Sufentanil were obtained by entering Sufentanil into the PubChem database, locating and downloading the schematic 2D Structure of Sufentanil (Figure 1), and uploading it to the Swiss Target Prediction database. Next, relevant predicted targets for pulmonary sepsis was obtained by entering Pulmonary sepsis into the GeneCards database. Then, these predicted targets of Sufentanil and Pulmonary sepsis were entered into the Venny 2.1.0 online tool, and the target intersection map of Sufentanil-Pulmonary sepsis and the common target genes of the two could be obtained. After that, the common targets of Sufentanil and Pulmonary sepsis were imported into the STRING database, and the species was qualified as “Homosapiens”, and the PPI network was mapped by Cytoscape 3.10.2 software. Finally, the common target data of Sufentanil and Pulmonary sepsis were imported into the GO-KEGG enrichment module developed by Wei Sheng Xin, and then the relevant targets were exported for the visualization of GO function and KEGG pathway enrichment as required.

### 2.6 Histopathological assay

Lung tissues were fixed in 4% paraformaldehyde solution for 3 days at 4°C, then dehydrated by ethanol with a concentration gradient, and then embedded in paraffin and sectioned (5 μm). The slices were stained with hematoxylin and eosin (G1120, Solarbio) for 5 min and 3 min, respectively, and the stained sections were dehydrated by ethanol and permeabilized by xylene, respectively, and sealed by neutral balsam. Lung slices were evaluated for pathological changes by optical microscope and ImageJ, and the results were recorded. To provide objective results, the scoring criteria were cited from the literature (Liu et al., 2016; Aziz et al., 2018), including alveolar wall thickness, alveolar structural disruption, and inflammatory cell infiltration, which were scored according to the scale of 0–4: 0 (normal), 1 (mild injury), 2 (moderate injury), 3 (significant injury), and 4 (serious injury) on a graded scale (Gao et al., 2022), and the three variables were summed to indicate organ damage.

### 2.7 ELISA

Saline 0.5 mL was introduced into lung tissue via trachea, and the thoracic cavity of mice was gently kneaded for about 20 s, and then bronchoalveolar lavage fluid (BALF) was collected. The above steps were repeated three times to collect enough BALF. The processed BALF was used to determine the levels of TNF-α(KE10002, Proteintech Group, Inc.), IL-1β(KE10003, Proteintech Group, Inc.), and IL-6(KE10007, Proteintech Group, Inc.) by an enzyme linked immunosorbent assay (ELISA) kit.

### 2.8 TUNEL staining

Cell apoptosis was detected in mouse lung tissue by terminal-deoxynucleoitidyl transferase mediated nick end labeling (TUNEL) staining (Millipore; Merck KGaA, Darmstadt, Germany) (Li et al., 2019). Lung tissue paraffin blocks were cut into 3 μm-thick slices. According to the instructions of the TUNEL staining kit, paraffin slices were deparaffinized with xylene, hydrated with gradient ethanol, and repaired with proteinase K (20 mg/L). The slices were washed three times with PBS. Then TUNEL reagent 50 μL was added to the slieces, which were incubated at 37°C for 1 h away from light, rinsed twice with PBS, sealed with DAPI-containing sealer, and observed and photographed under a microscope (Sigma-Aldrich, St. Louis, MO).

### 2.9 Western blotting

Lung tissue samples from each group of mice were crushed and lysed by RIPA lysis buffer (strong) for 2 h. The supernatant obtained after centrifugation was subjected to protein quantification by BCA Protein Assay Kit. Protein samples 35 μg were taken, and proteins were separated by SDS-PAGE, and then transferred to PVDF membrane, which was closed by using 5% skim milk powder at room temperature for 2 h. Finally, rabbit anti-rat JAK-2 (AF1489,Beyotime Biotechnology), p-JAK2 (AF1486, Beyotime Biotechnology), STAT3 (AF1492, Beyotime Biotechnology) and p-STAT3 (AF1276, Beyotime Biotechnology) antibody (primary antibody) was added sequentially, and the membrane was incubated overnight at 4°C, and washed with PBS. Next, the membrane was incubated with secondary antibody with HRP labeling at room temperature for 1 h and then washed. Protein bands were observed by the ECL reagent (PE0010, Solarbio), exposed in dark room, and analyzed for gray value. The relative expression of the target proteins was calculated with GAPDH as an internal reference.

### 2.10 Statistical analysis

All the results were subject to at least three independent experiments. All data were expressed as xˉ ± s, and statistical analysis was performed using GraphPad Prism 8.0 statistical software. The group differences were examined using one-way ANOVA, followed by Tukey’s post-hoc multiple comparison test. p < 0.05 was considered to indicate a statistically significant difference.

## 3 Results

### 3.1 Screening of targets of sufentanil for the treatment of septic ALI

Screening of targets of Sufentanil for the treatment of septic ALI A total of 110 predicted targets of Sufentanil were obtained from the Swiss Target Prediction database (Table 1).

**TABLE 1:** Potential effect targets of Sufentanil.

HIPK4	CDK2	DPP7	STK38	IGF1R	PSENEN	MAK	FLT1	P2RX7	CCNA1
GRIN1	GRK7	MAST1	MAP2K1	PDE10A	ACVRL1	FNTB	FYN	CHRNB2	MAP3K13
FLT3	PSEN2	HTR2A	DRD1	ADRA2A	CCR2	SLC18A2	FNTA	SSTR3	CACNA1G
NTRK2	ICK	SCN5A	OPRD1	PRKCQ	MEN1	DRD4	CDK8	MET	KDR
NTRK3	GSK3B	ABCB1	SLC6A11	CHRNA4	OPRM1	CALCRL	PDE9A	TRPM8	CHRNA3
UTS2R	GRIN2B	HTR2C	ADRA2B	MDM2	PIK3C2G	CDKL3	PRKCD	STK39	CSF1R
SBK1	EGFR	OPRK1	ERBB2	CYP2J2	NOS2	CACNA1I	CCR1	HUNK	INSR
NCSTN	PRKCG	PSEN1	SLC6A12	CDK13	RAF1	MAP3K15	PGGT1B	CASR	ROS1
PRKCE	APH1B	CYP51A1	MAPK14	APH1A	CDKL5	DPP9	OPRL1	RET	HTR2B
PRKCA	CCNA2	TAOK2	OXSR1	CHRNB4	LRRK2	TGFBR1	SLC6A5	JAK1	DPP8
ADRA2C	PRKCB	ERNI	SLC6A13	ABL1	MAP3K6	JAK2	SRC	PDE1C	MAP3K20

### 3.2 Intersection targets of sufentanil and pulmonary sepsis

**FIGURE 2:**
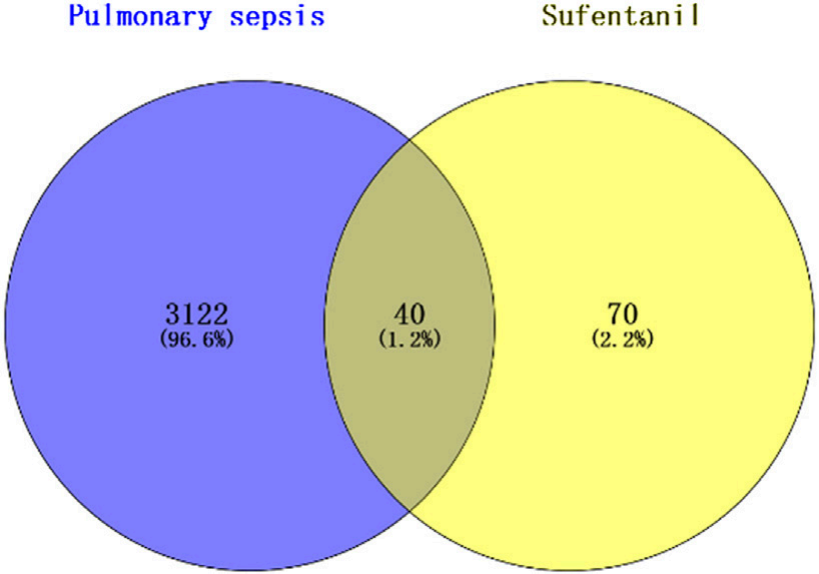
Intersection of target sites for sufentanil and pulmonary sepsis.

A total of 3,162 targets related to pulmonary sepsis were obtained from the GeneCards database, and the intersection of the predicted targets of Sufentanil and targets related to pulmonary sepsis was obtained by the Venny 2.1.0 online tool, resulting in a total of 40 potential targets for the treatment of pulmonary sepsis with Sufentanil (Figure 2).

### 3.3 Network the PPI

**FIGURE 3:**
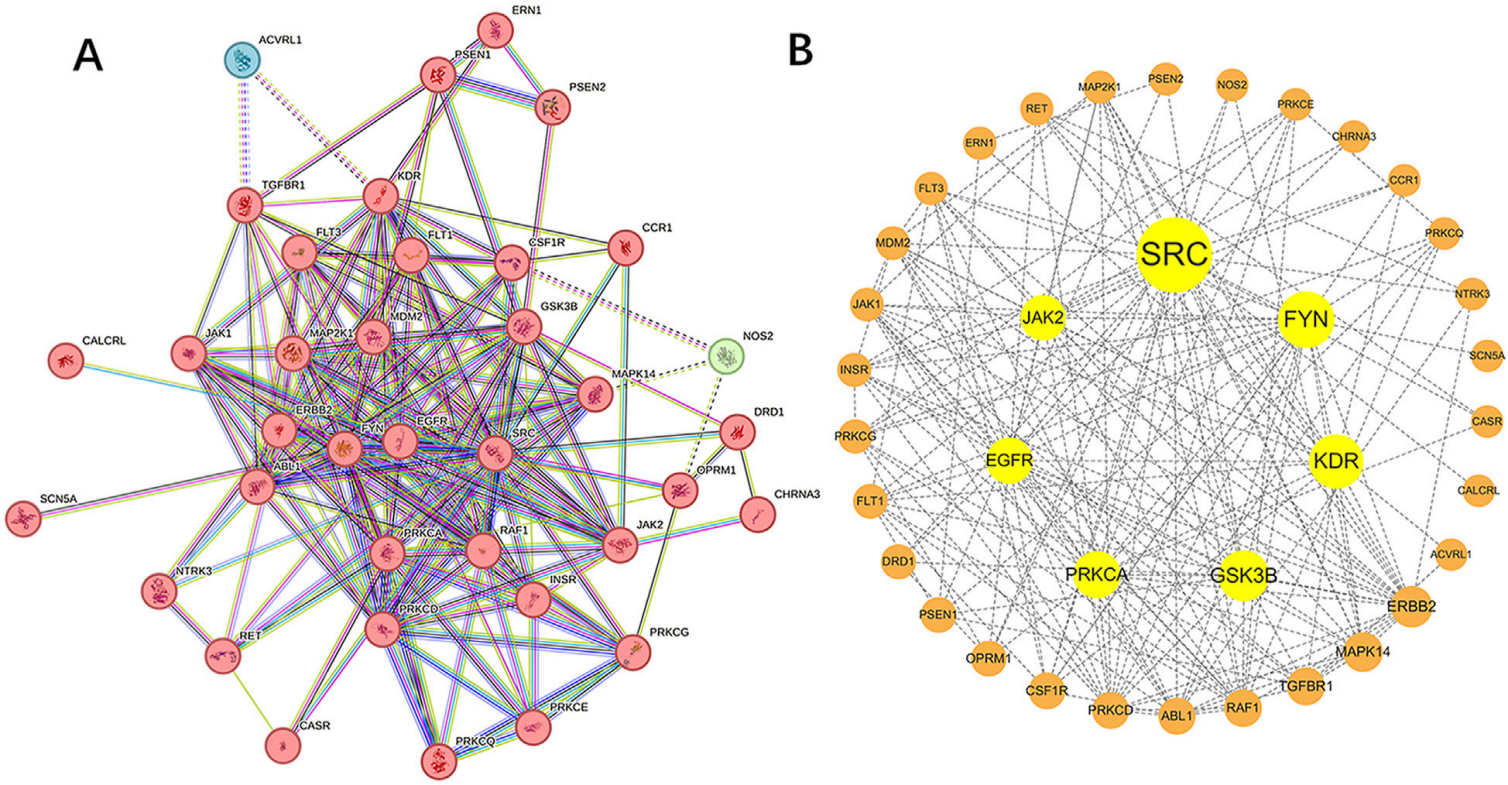
Target proteins of sufentanil in pulmonary sepsis. **(A)** PPI network diagram for the intersection of sufentanil and pulmonary sepsis. **(B)** target protein network.

In the STRING database, we associated 40 target genes according to function and physical protein, set the minimum required interaction score to medium confidence (0.400), and then hid the unconnected nodes in the network. Finally, 37 target genes were divided into 3 clusters, of which 2 clusters had 1 target gene each. Network of Sufentanil and pulmonary sepsis was constructed by STRING 11.0 (Figure 3A), and then the targets in the PPI network were imported into the Cytoscape software to arrange the PPI target proteins of the two (Figure 3B). Betweenness Centrality with no weights is selected for analysis, a larger value indicates stronger target interaction. According to the PPI diagram, the potential targets of Sufentanil for pulmonary sepsis included JAK2, SRC, FYN, KDR, GSK3B, PRKCA and EGFR. Among them, JAK2 has a high Betweenness Centrality of 58.2330891330891.

### 3.4 GO functional enrichment analysis

**FIGURE 4:**
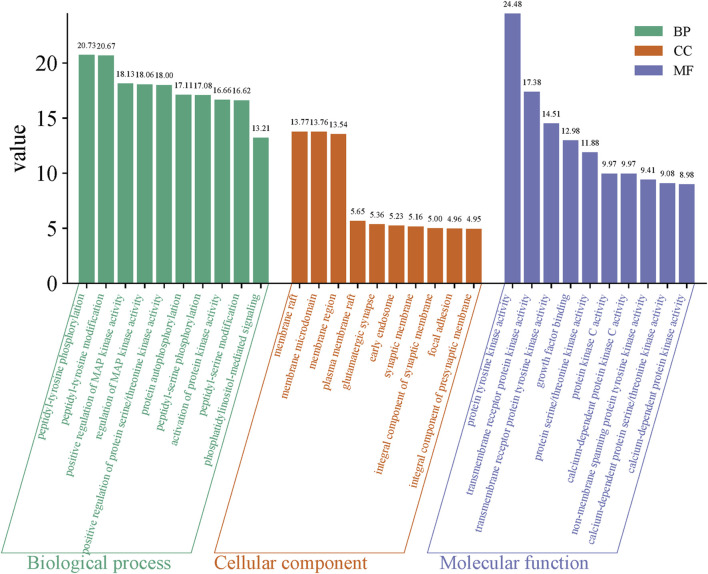
Gene ontology (GO) functional enrichment analysis.

GO functional enrichment analysis of the intersection targets of Sufentanil and pulmonary sepsis (Figure 4) revealed that the effect target genes were mainly enriched in 1,483 BPs, primarily peptidyl-tyrosine phosphorylation, peptidyl-tyrosine modification, positive regulation of MAP kinase activity, regulation of MAP kinase activity, positive regulation of protein serine/threonine kinase activity, protein autophosphorylation and peptidyl-serine phosphorylation; 84 CCs, primarily membrane raft, membrane microdomain, membrane region, plasma membrane raft, glutamatergic synapse, early endosome, synaptic membrane and integral component of synaptic membrane; and 125 MFs, primarily protein tyrosine kinase activity, transmembrane receptor protein kinase activity, transmembrane receptor protein tyrosine kinase activity, growth factor binding, protein serine/threonine kinase activity and protein kinase C activity and calcium-dependent protein kinase C activity. This suggested that Sufentanil may exert its therapeutic effects on pulmonary sepsis through the regulation of biological processes by these molecular functions.

### 3.5 KEGG pathway enrichment analysis

**FIGURE 5:**
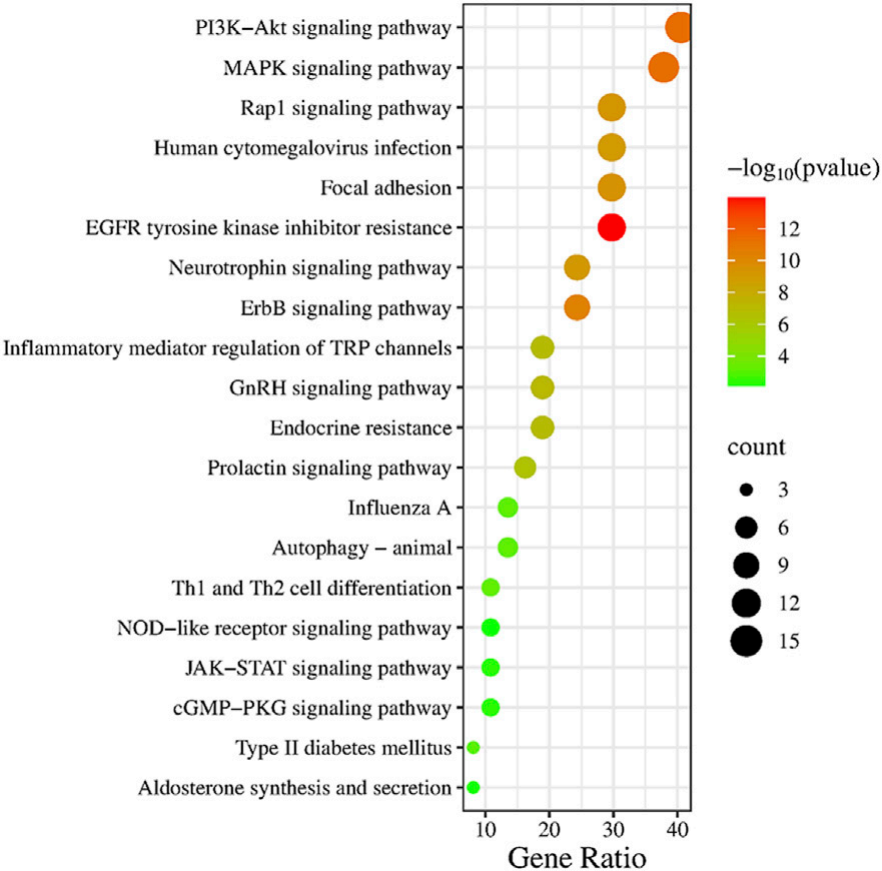
KEGG pathway enrichment analysis.

According to the above analysis results, we conducted KEGG pathway enrichment analysis for intersection genes. Through the KEGG pathway enrichment analysis of intersection genes, 20 representative signaling pathways with p < 0.05 were identified (Figure 5), including JAK-STAT signaling pathway, EGFR tyrosine kinase inhibitor resistance, PI3K-Akt signaling pathway, MAPK signaling pathway, ErbB signaling pathway, Focal adhesion, Rap1 signaling pathway, Prolactin signaling pathway, Influenza A, Type II diabetes mellitus, cGMP-PKG signaling pathway, NOD-like receptor signaling pathway and Aldosterone synthesis and secretion. Among them, JAK-STAT signaling pathway, as a signaling pathway closely associated with inflammation, plays a crucial role in the progression of pulmonary sepsis and is closely related to the core targets screened above, including JAK2, SRC, FYN and KDR. According to relevant literature and key target prediction, as well as KEGG pathway enrichment analysis, the JAK2-STAT3 signaling pathway was selected for preliminary experimental validation.

### 3.6 Effect of sufentanil on the pathological morphology of mouse lung tissue

**FIGURE 6:**
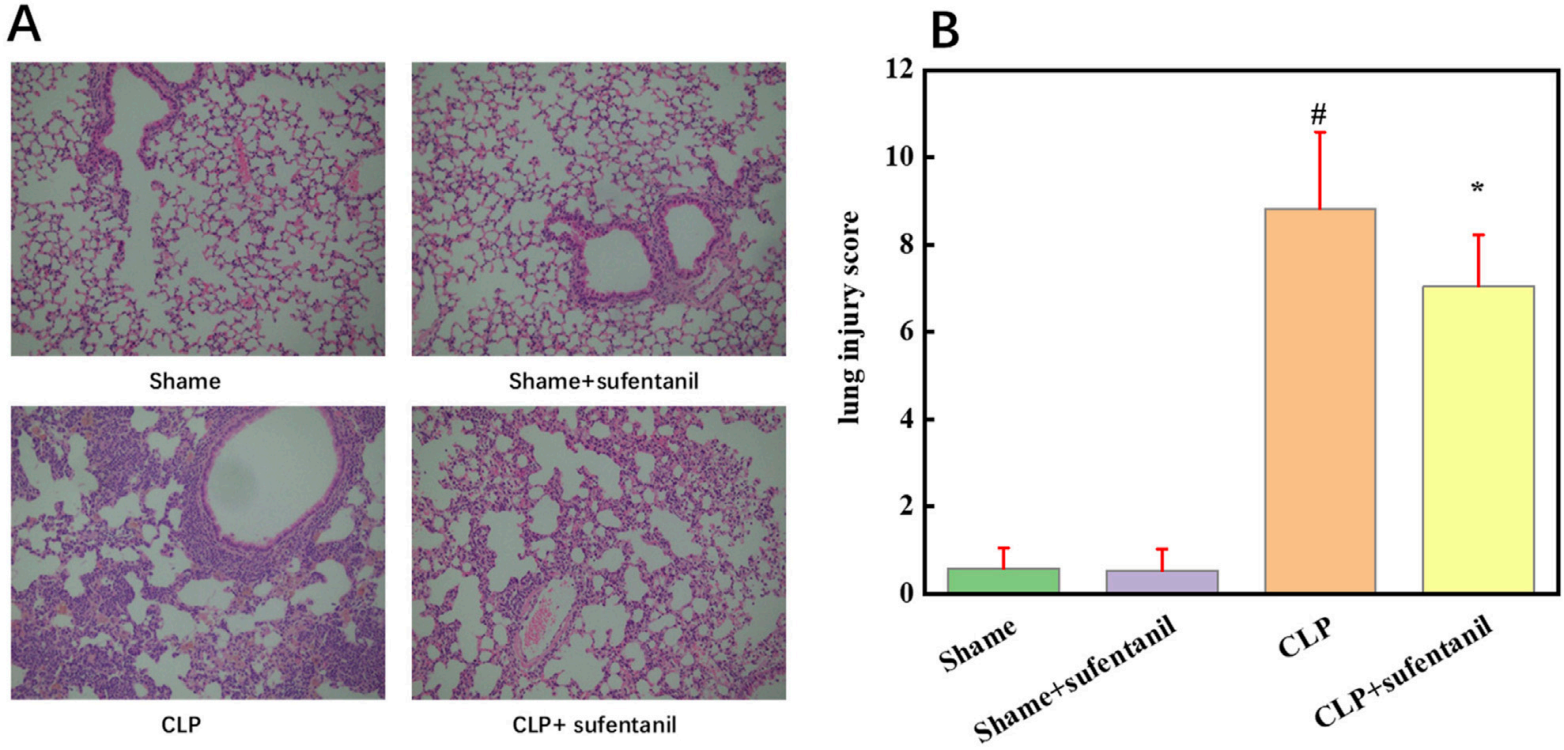
Sufentanil Improves Lung Tissue Pathobiology and Damage in Mice Across All Groups. **(A)** Effects of sufentanil on the histomorphology of mouse lung tissue. H&E staining of lung tissue. Magnification, × 400. **(B)** Lung injury scores in mice for each group. Data are shown as the mean ± standard error of mean (n = 10). #p < 0.01 vs. shame; *p < 0.01 vs. CLP. All operations were done in triplicate.

As shown by HE staining, lung tissue structure of the Sham group and the Sham + Sufentanil group was intact, while alveolar wall of the CLP group was significantly thickened and alveolar structure was severely damaged, resulting in a significant reduction in normal alveoli as well as severe inflammatory cell infiltration. However, morphological changes in the lung tissue of mice in the CLP + Sufentanil group were more significantly improved compared to the CLP group (Figure 6A). In addition, the scores of pathological changes in lung tissue were calculated, which showed the scores of the control group and the control + sufentanil group were (0.58 ± 0.47) and (0.54 ± 0.49), respectively. The score of the CLP group is significantly increased (8.82 ± 1.76), and after treatment with Sufentanil, the score of the sufentanil group is significantly decreased (7.05 ± 1.17) (Figure 6B). In summary, Sufentanil was probably effective in alleviating lung tissue injury and inflammatory response in septic mice.

### 3.7 Effect of sufentanil on apoptosis in lung tissues of mice

**FIGURE 7:**
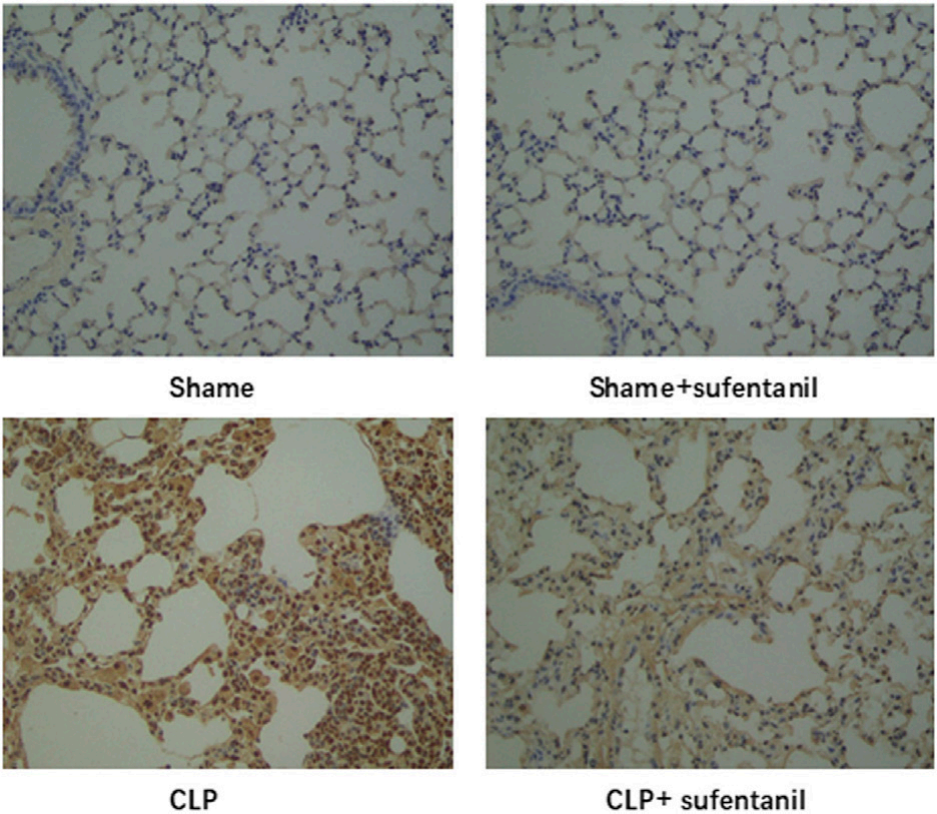
Effects of sufentanil on apoptosis in lung tissues of mice in each group.

As is shown, there was no significant difference in apoptosis between the Sham group and the Sham + Sufentanil group, and the number of apoptotic cells was significantly higher in the CLP group compared with the Sham group. However, apoptotic cells in the CLP group decreased significantly after treatment with Sufentanil (Figure 7). The results showed that Sufentanil had the effect of reducing cell apoptosis in lung tissues of CLP model mice with sepsis.

### 3.8 Effect of sufentanil on BALF levels of TNF-α, IL-1β and IL-6 in different groups of mice

**FIGURE 8:**
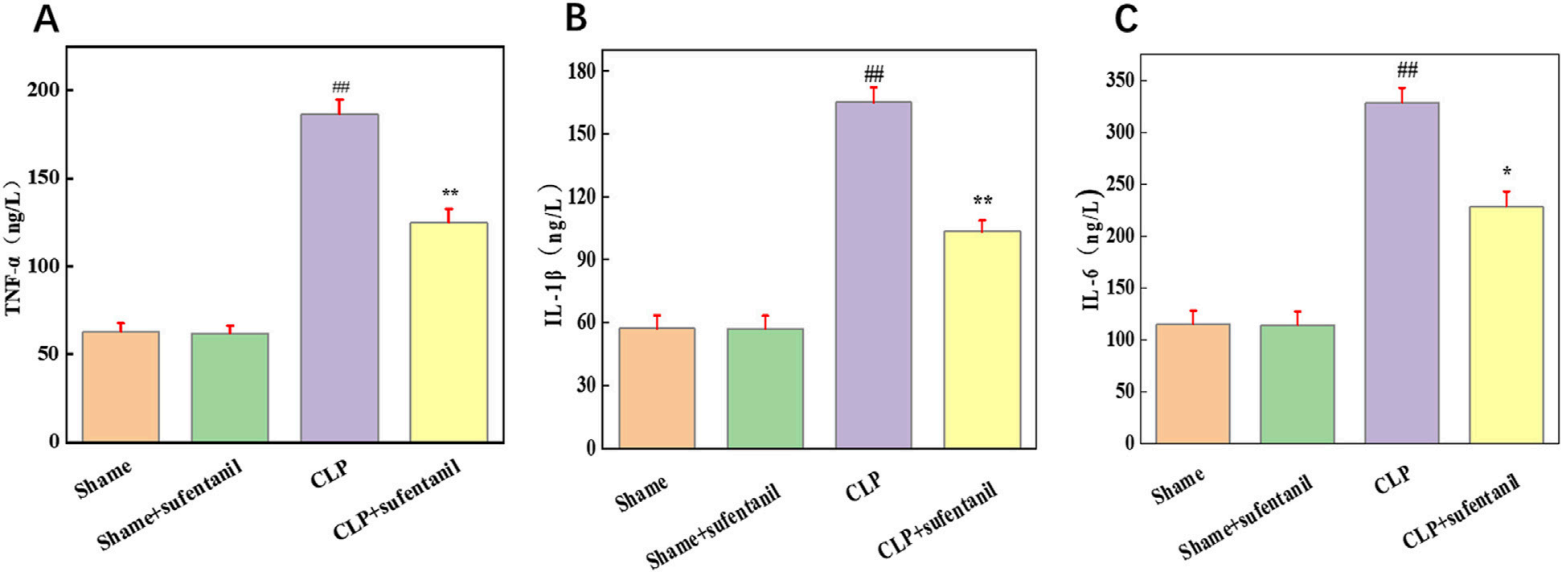
Sufentanil attenuates inflammatory response in mice across all groups. **(A)** TNF-α, **(B)** IL-6, **(C)** IL-1β. Data are shown as the mean ± standard error of mean (n = 10). ##p < 0.001 vs. shame; **p < 0.01, *P < 0.05 vs. CLP. All operations were done in triplicate.

TNF-α, IL-1β and IL-6 are pro-inflammatory cytokines that play a central role in the pathogenesis of sepsis. To determine the effect of Sufentanil on inflammation, we measured the levels of inflammatory cytokines TNF-α (Figure 8A), IL-1β (Figure 8B) and IL-6 (Figure 8C). As is shown, there was no significant difference in the levels of inflammatory cytokines between the Sham group and the Sham + Sufentanil group, which remained at low levels, TNF-α (62.86 ± 4.76 ng/L vs. 61.90 ± 4.48 ng/L, IL-1β (57.14 ± 6.43 ng/L vs. 56.96 ± 6.27 ng/L), and IL-6 (115.16 ± 12.86 ng/L vs. 114.28 ± 12.98 ng/L). Compared with these two groups, inflammatory cytokines were significantly elevated in the CLP group, TNF-α (186.67 ± 8.19 ng/L), IL-1β (165.13 ± 7.14 ng/L) and IL-6 (328.57 ± 14.28 ng/L). However, the CLP group demonstrated a decrease in inflammatory cytokine levels after treatment with Sufentanil, TNF-α(124.76 ± 7.90 ng/L), IL-1β(103.57 ± 5.18 ng/L) and IL-6 (228.57 ± 14.29 ng/L). The results suggested that Sufentanil may alleviate the inflammatory response in septic mice.

### 3.9 Effect of sufentanil on the expression of JAK2, p-JAK2, STAT3, and p-STAT3 proteins in lung tissue of mice in various groups

**FIGURE 9:**
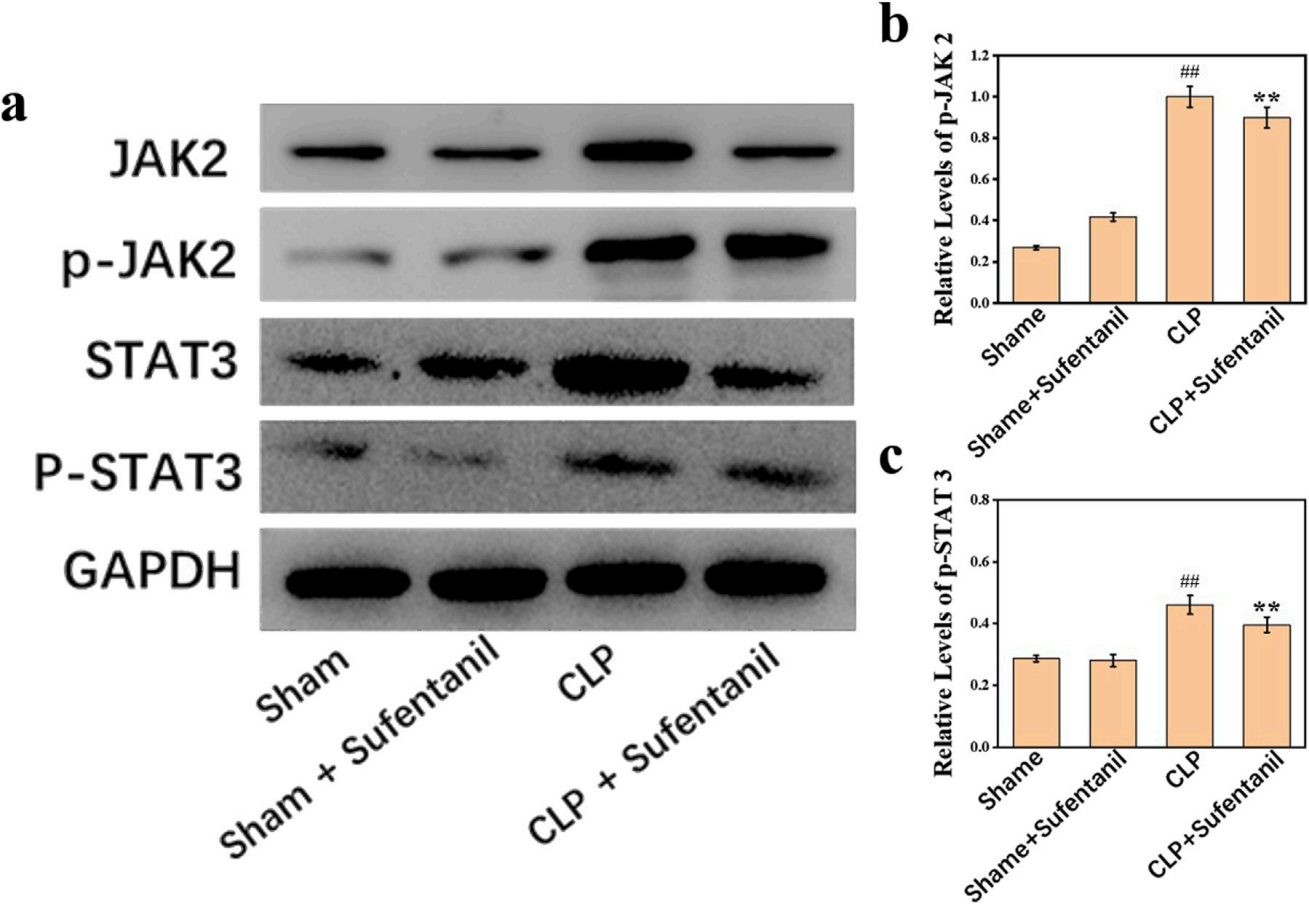
**(A)** Expression of JAK2, **(B)** p-JAK2, STAT3, and **(C)** p-STAT3 proteins in lung tissues of mice in each group following treatment with sufentanil. Data are shown as the mean ± standard error of mean (n = 10). ##p < 0.001 vs. shame; **p < 0.01 vs. CLP. All operations were done in triplicate.

There was no significant difference in JAK2, P- JA2, STAT3 and P-STAT3 protein expression between the Sham and Sham + Sufentanil groups (Figure 9A). However, JAK2, P- JA2, STAT3 and P-STAT3 protein expression was significantly elevated in the CLP group; but a significant decrease in JAK2, P- JA2, STAT3 and P-STAT3 protein expression could be found in the CLP group after treatment with Sufentanil (Figures 9B, C).

### 3.10 Effects of sufentanil on expression of apoptosis-related proteins in lung Bax, Bcl-2, cleaved caspase 3 in each group of mice

**FIGURE 10:**
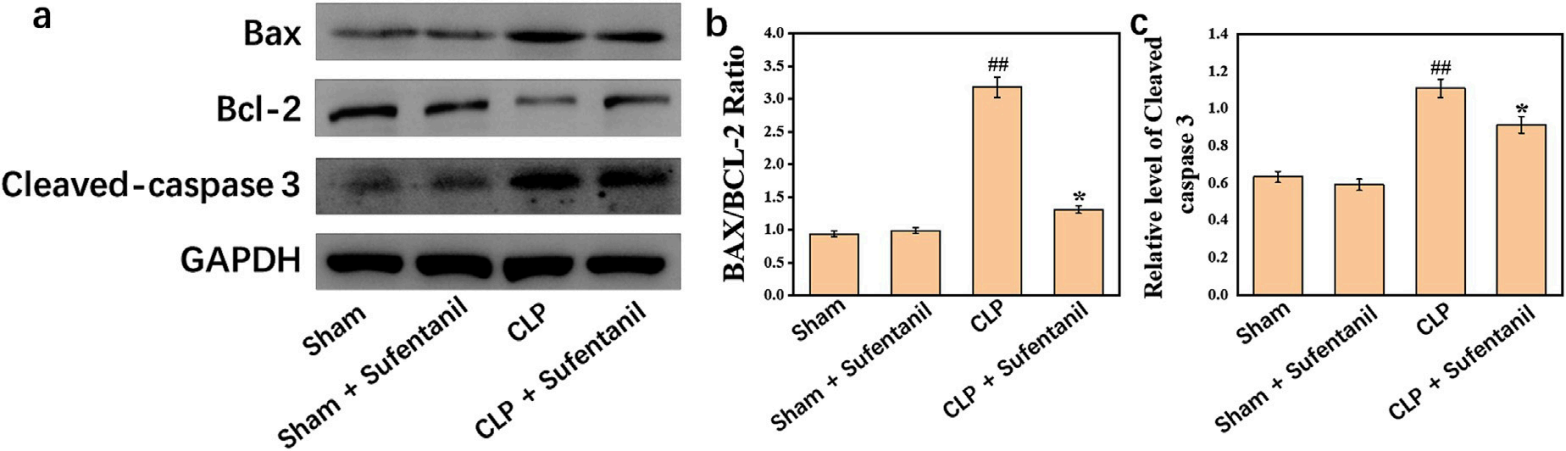
**(A)** Expression of bax, **(B)** bcl-2 and **(C) **cleaved caspase 3 proteins in lung tissues of mice in each group following treatment with sufentanil. Data are shown as the mean ± standard error of mean (n = 10). ##p < 0.001 vs. shame; *p < 0.01 vs. CLP. All operations were done in triplicate.

There were no significant differences in the expression of Bax, Bcl-2, Cleaved caspase 3 between Sham group and Sham + Sufentanil group (Figure 10A). However, in the CLP group, the expressions of pro-apoptotic Bax and Cleaved caspase 3 were significantly increased, and the anti-apoptotic Bcl-2 protein was significantly decreased. However, in the CLP group treated by Sufentanil, the expression of pro-apoptotic Bax and Cleaved caspase 3 was significantly decreased compared with the CLP group, and the expression of anti-apoptotic Bcl-2 protein was increased compared with the CLP group (Figures 10B, C) (all P < 0.05). Therefore, this study demonstrated that sufentanil can inhibit apoptosis associated with lung injury in sepsis.

## 4 Discussion

Septic ALI is a life-threatening organ dysfunction resulting from diffuse alveolar epithelial cell and pulmonary capillary endothelial cell damage due to over-response to infection caused by a variety of causative factors through multiple mechanisms, and is often accompanied by respiratory failure (Fowler et al., 2019; Soares et al., 2012). Sepsis ALI is often characterized by an overwhelming inflammatory response, an overactivated inflammatory response that results in pathological damage to the alveolar epithelium and vascular endothelium. Inflammation plays a crucial role in ali. At the same time, we found that sufentanil is commonly used in the treatment of patients with sepsis. Pain in sepsis patients hospitalized in intensive care is one of the causes of their anxiety and agitation (Bulic et al., 2017). Therefore, analgesia is beneficial to moderately reduce the incidence of anxiety, agitation, and delirium in sepsis patients (Bateman et al., 2016), as well as to shorten the length of intensive care hospital stay and save medical costs (Barr et al., 2013). Studies have shown that the use of sufentanil in ICU patients can also reduce lung damage caused by mechanical ventilation, and the advantages of sufentanil compared to fentanyl are stronger analgesic effect, less physiological interference, and a lower incidence of adverse reactions (Yang et al., 2014). Recent studies have shown that sufentanil has positive anti-inflammatory and anti-tumor effects (Lu et al., 2022; Liu et al., 2023; Li et al., 2023). With the rise of sufentanil in the treatment of sepsis, we chose CLP mouse model to study it. Firstly, 44 core targets of sufentanil in the treatment of acute lung injury in sepsis were discovered through network pharmacology, and key targets such as JAK2, SRC and EGFR were screened through PPI network construction. They are all involved in regulating cell signaling and gene expression, influencing inflammation and immune responses in sepsis (Hamers et al., 2015). Among them, SRC can regulate the behavior of immune cells by activating multiple signaling pathways (Rimar et al., 2021). Activation of SRC may lead to cell necrosis, which is closely related to the inflammatory response, and the SRC inhibitor PP2 is known to inhibit TNF-α-induced necrosis without inducing apoptosis (Li et al., 2020). In addition, SRC may be involved in the clearance of pathogens that cause infection in the body (Chowdhury et al., 2019). Interestingly, SRC kinase can directly phosphorylate EGFR sites, which not only enhances the kinase activity of EGFR itself, but also promotes the binding of other signaling molecules to EGFR, thereby amplifying the signaling effect of EGFR (Biscardi et al., 1999; Wu et al., 2002). EGFR consists of three domains: an intracellular domain with tyrosine kinase regions, a transmembrane domain, and a cysteine-rich extracellular domain (Holbro and Hynes, 2004). Activation of the EGFR signaling pathway regulates cell proliferation and inflammatory processes (Rayego-Mateos et al., 2018). JAK2, a non-receptor tyrosine kinase, is one of the JAK subtypes. Activation and phosphorylation of JAK2 by proinflammatory factors can further induce the phosphorylation of STAT3, resulting in the expression of multiple proinflammatory factors (He et al., 2022; Xu et al., 2020). It has been reported that this signaling pathway appears to provide therapeutic targets for inflammation, coagulation, and lung injury in mouse models of *in vivo* and *in vitro* sepsis (Lu et al., 2023). In addition, our KEGG pathway enrichment analysis showed that sufentanil may show protective effects on acute lung injury caused by sepsis through JAK-STAT, PI3K-Akt, and MAPK signaling pathways. JAK2/STAT3 signaling pathway is an important pathway involved in cell growth, survival, immune regulation and various physiological processes after the activation of cytokine receptors and growth factor receptors. Ulinastatin (Wu et al., 2019b), pterostilbene (Xue and Li, 2020), Taxifolin (Shen et al., 2022) and Maresin1 (Wang et al., 2020) all showed the effect of inhibiting JAK2/STAT3 signaling pathway to reduce inflammation in sepsis and protect lung injury. Combined with sufentanil’s target protein in acute lung injury induced by sepsis, comprehensive analysis shows that the JAK2/STAT3 signaling pathway is strongly correlated with the application of sufentanil in acute lung injury induced by sepsis. In order to determine whether sufentanil acts through the JAK2-STAT3 signaling pathway and its specific mechanism, We established a mouse model of sepsis. Based on HE staining and ELISA detection, we found that compared with the CLP group, the area of lung tissue damage and the levels of inflammatory factors TNF-α, IL-1β and IL-6 in the Sufentanil treated group were significantly improved. Sufentanil was shown to reduce the level of ALI inflammation. Studies have shown that the JAK2/STAT3 signaling pathway is activated by cytokine receptors and growth factor receptors (Clere-Jehl et al., 2020). We have confirmed that sufentanil can reduce the level of inflammatory factors in ALI. Next, we extracted lung tissue proteins from mice and improved Western blotting experiments. The results showed that the expression of p-JAK2 and p-STAT3 decreased significantly after sufentanil treatment. These results indicate that sufentanil can inhibit JAK2-STAT3 signaling pathway related inflammation by reducing JAK2 and STAT3 phosphorylation.

Apoptosis and inflammation do not occur in isolation in ALI and there is a complex interaction between them. Apoptotic cells can release damage-related molecular patterns (DAMPs) that activate immune cells and exacerbate inflammatory responses (Nedeva, 2021). At the same time, inflammatory mediators such as TNF-α and IL-1β can promote apoptosis, forming a vicious cycle in which inflammation promotes apoptosis and apoptosis further exacerbates inflammation (O'Brien et al., 2007). The results of our TUNEL experiment and Western blotting experiment also confirmed that Sufentanil treatment can reduce the apoptosis of lung tissue cells induced by sepsis in CLP model mice. Sufentanil may improve the inflammatory response by inhibiting inflammatory mediators and JAK2/STAT3 signaling pathway, and at the same time reduce the level of apoptosis, and to some extent interrupt the vicious cycle of mutual promotion of inflammation and apoptosis. Sufentanil has a positive protective effect on the progression of acute lung injury in sepsis.

## 5 Conclusion

In this study, network pharmacology was used to predict the possible key targets and signaling pathways of sufentanil in the protection of sepsis lung, and preliminary validation was conducted through animal experiments. The results showed that Sufentanil may reduce cell inflammation and apoptosis through JAK2-STAT3 signaling pathway, and then protect lung injury in sepsis. Sufentanil can not only produce analgesic effect by acting on the μ-opioid receptors in the brain, but also reduce the inflammatory response of patients with pulmonary sepsis, protect the lung and improve the quality of life of patients with pulmonary sepsis, providing further support for the application of sufentanil in the treatment of patients with acute lung injury caused by sepsis, and may be used in the daily treatment of patients with pulmonary sepsis. There are still shortcomings in this study: (1) The mechanism of sufentanil’s improvement of lung injury in sepsis mice is relatively complex, and whether sufentanil acts on upstream and downstream factors of JAK2-STAT3 pathway and other pathways needs to be further confirmed. (2) This study only verified that sufentanil may protect lung injury in sepsis through JAK2-STAT3 signaling pathway in animal experiments. However, its effectiveness needs to be further verified through cell experiments and other forms. (3) If sufentanil is applied in clinic, a large number of experiments need to be conducted to verify its safety and effectiveness.

## Data Availability

The original contributions presented in the study are included in the article/Supplementary Material, further inquiries can be directed to the corresponding author.
