# Supplementary figures and images for: Protective effect and mechanism of Sufentanil on acute lung injury in septic mice
Source: Front Pharmacol. 2025 Jan 16;15:1514602. doi: 10.3389/fphar.2024.1514602 (PMC11780379; doi:10.3389/fphar.2024.1514602)

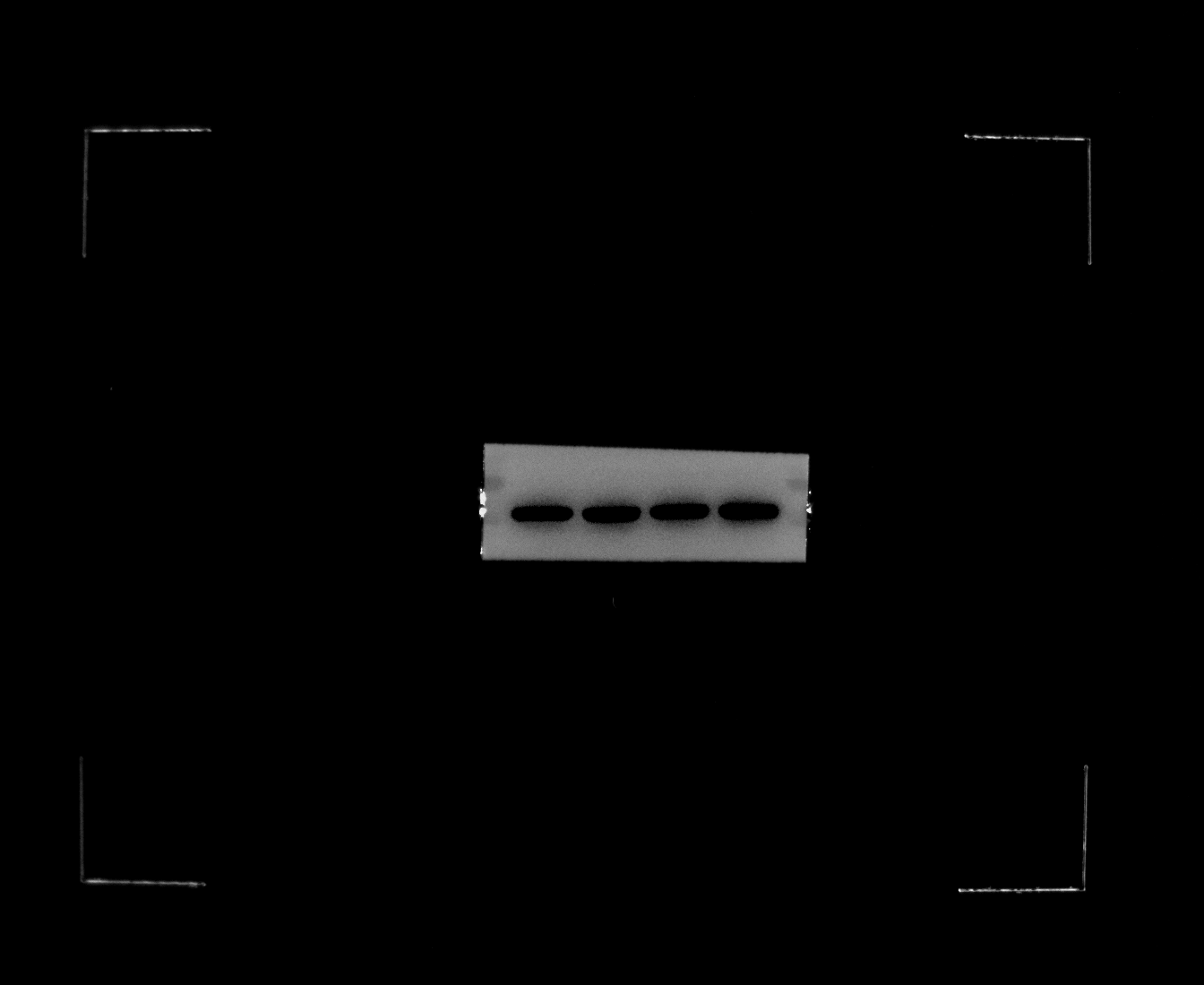

Supplement: Supplementary file 1 [file DataSheet1.zip › Supplementary Material-The original image files for the blots/GAPDH.tif]

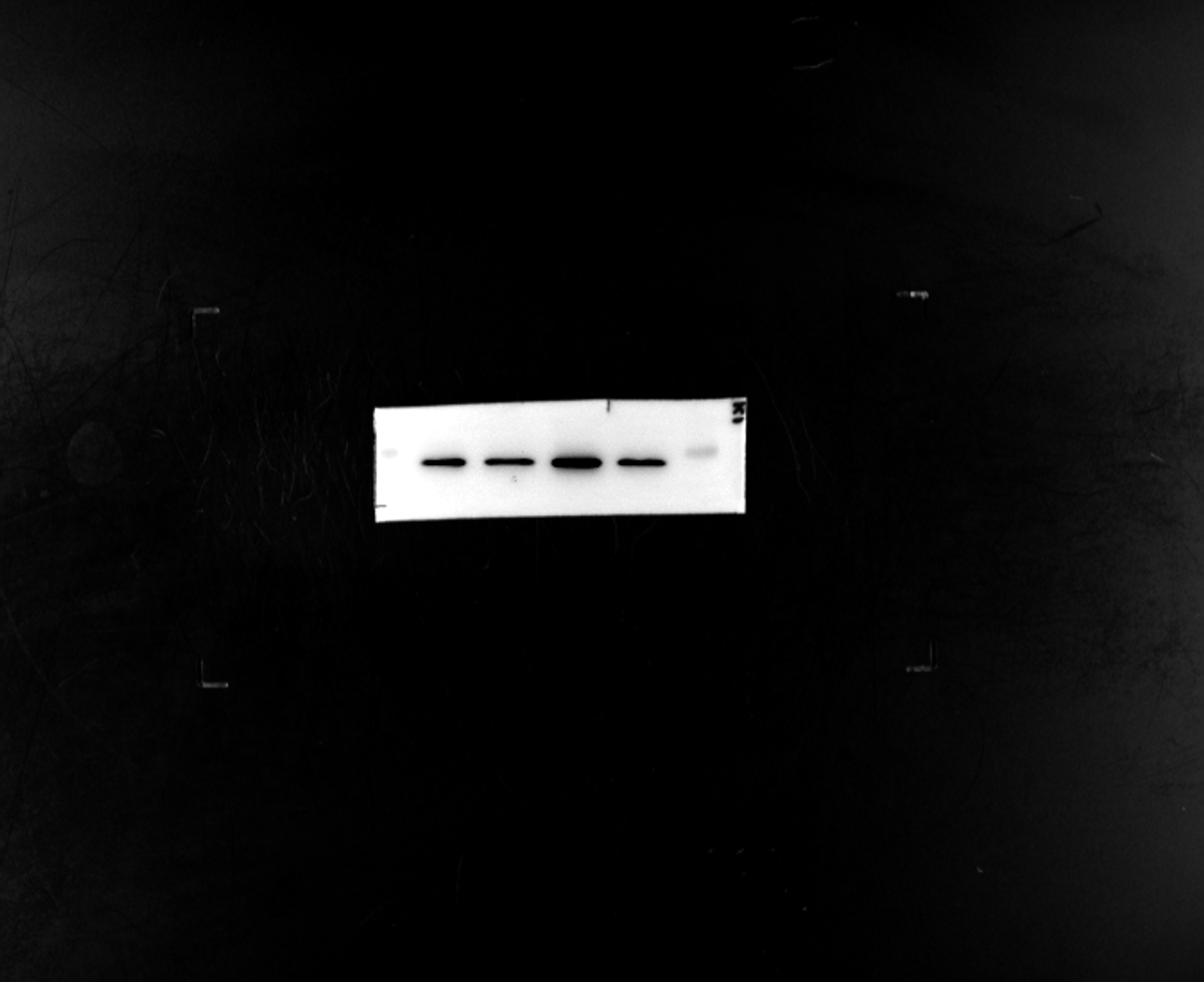

Supplement: Supplementary file 1 [file DataSheet1.zip › Supplementary Material-The original image files for the blots/JAK2.tif]

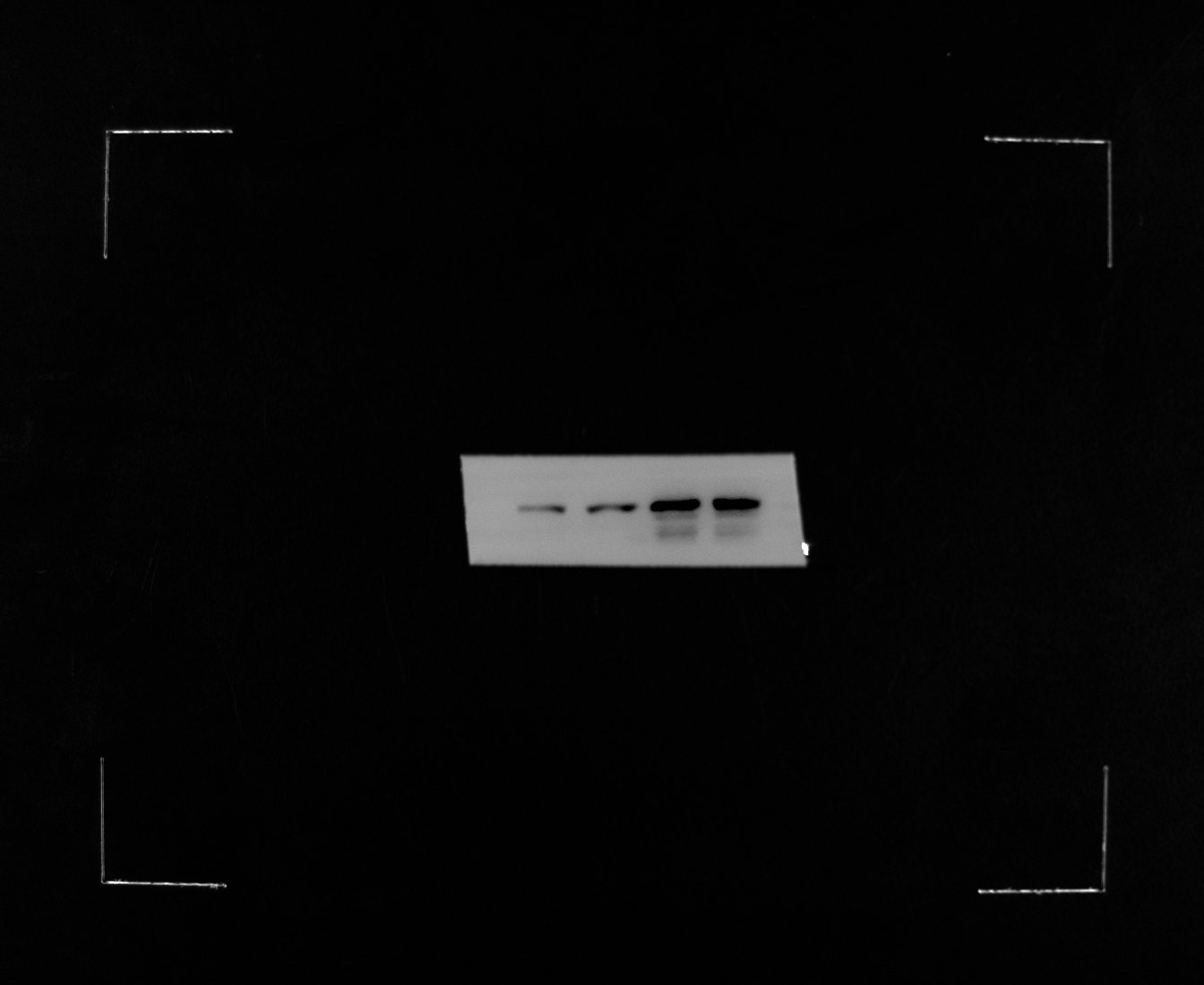

Supplement: Supplementary file 1 [file DataSheet1.zip › Supplementary Material-The original image files for the blots/P-JAK2.tif]

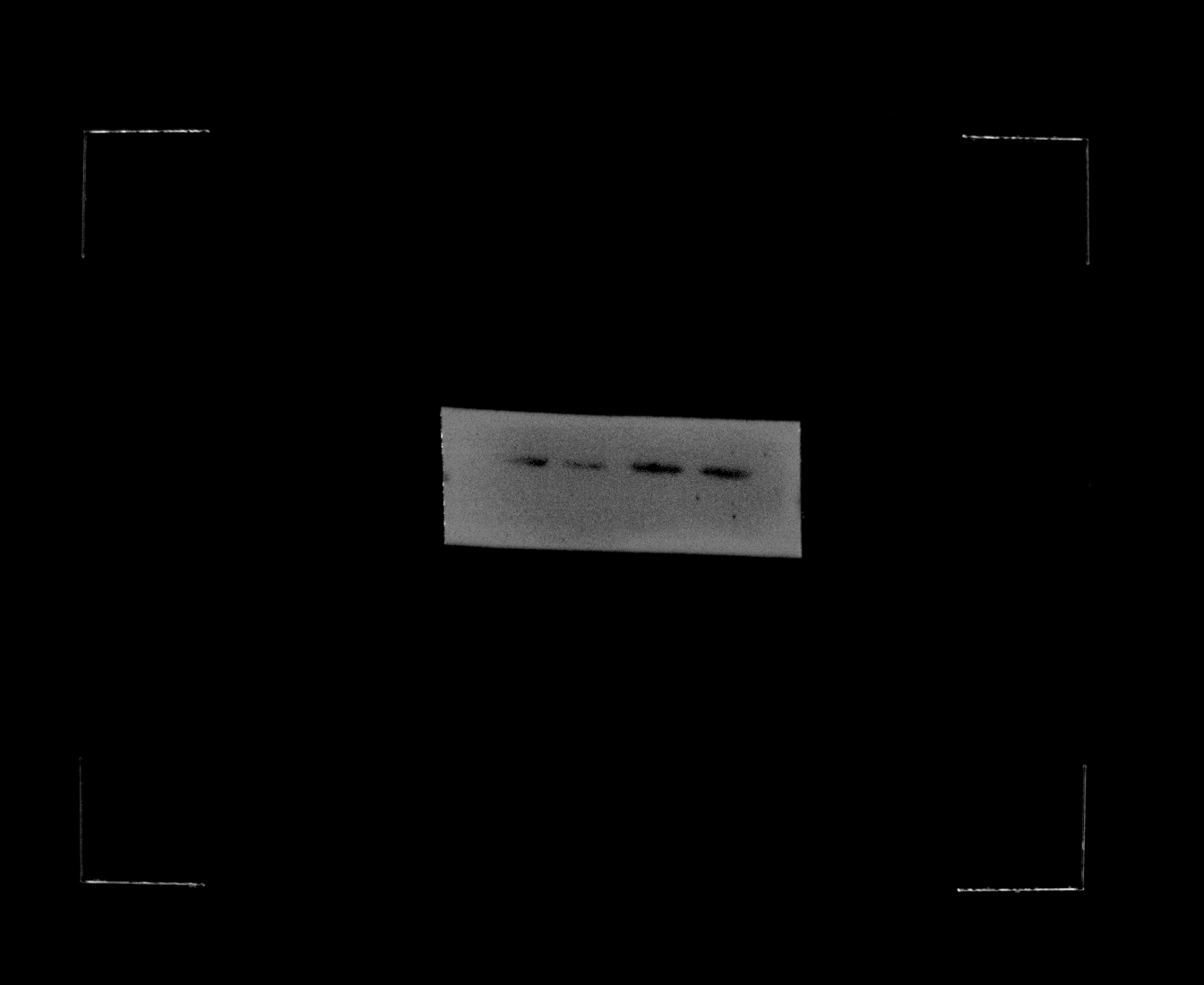

Supplement: Supplementary file 1 [file DataSheet1.zip › Supplementary Material-The original image files for the blots/P-STAT3.tif]

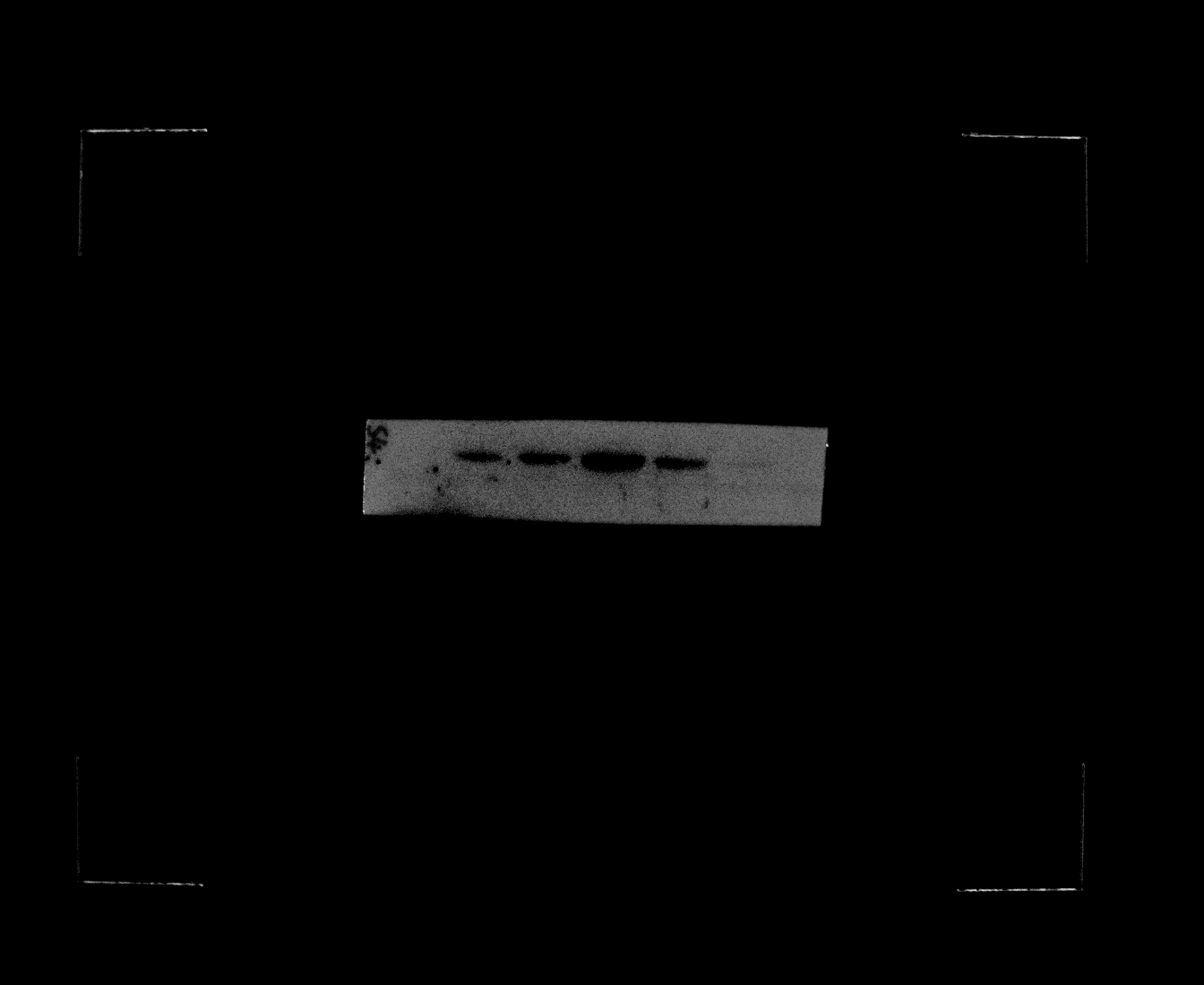

Supplement: Supplementary file 1 [file DataSheet1.zip › Supplementary Material-The original image files for the blots/STAT3.tif]
